# Low-risk polycythemia vera treated with phlebotomies: clinical characteristics, hematologic control and complications in 453 patients from the Spanish Registry of Polycythemia Vera

**DOI:** 10.1007/s00277-022-04963-z

**Published:** 2022-08-30

**Authors:** Ana Triguero, Alexandra Pedraza, Manuel Pérez-Encinas, María Isabel Mata-Vázquez, Patricia Vélez, Laura Fox, Montse Gómez-Calafat, Regina García-Delgado, Mercedes Gasior, Francisca Ferrer-Marín, Valentín García-Gutiérrez, Anna Angona, María Teresa Gómez-Casares, Beatriz Cuevas, Clara Martínez, Raúl Pérez, José María Raya, Lucía Guerrero, Ilda Murillo, Beatriz Bellosillo, Juan Carlos Hernández-Boluda, Cristina Sanz, Alberto Álvarez-Larrán, On behalf of the MPN Spanish Group (GEMFIN)

**Affiliations:** 1 Hospital Clinic de Barcelona, Barcelona, Spain; 2 Complejo Hospitalario Universitario de Santiago, Santiago de Compostela, Spain; 3 Hospital Costa del Sol, Marbella, Spain; 4 Hospital del Mar, Barcelona, Spain; 5 Hospital Universitario Vall d’Hebron, Hematología Experimental, Vall d’Hebron Institute of Oncology (VHIO), Barcelona, Spain; 6 Hospital Clínico Universitario de Valencia, Valencia, Spain; 7 Hospital Universitario Virgen de La Victoria, Málaga, Spain; 8 Hospital Universitario La Paz, Madrid, Spain; 9 Hospital Universitario Morales-Meseguer, CIBERER-UCAM, Murcia, Spain; 10 Hospital Universitario Ramón Y Cajal (IRYCIS), Madrid, Spain; 11 ICO Girona-Hospital Josep Trueta, Girona, Spain; 12 Hospital Universitario de Gran Canaria Dr. Negrín, Las Palmas de Gran Canaria, Spain; 13 Hospital Universitario de Burgos, Burgos, Spain; 14 Hospital de La Santa Creu I Sant Pau, Barcelona, Spain; 15 Hospital Universitario Clínico Virgen de La Arrixaca, Murcia, Spain; 16 Hospital Universitario de Canarias, Tenerife, Spain; 17 Hospital Rio Carrión, Palencia, Spain; 18 Hospital General San Jorge, Huesca, Spain

**Keywords:** Polycythemia vera, Low-risk, Phlebotomies, Thrombosis, Myelofibrosis

## Abstract

Hematological control, incidence of complications, and need for cytoreduction were studied in 453 patients with low-risk polycythemia vera (PV) treated with phlebotomies alone. Median hematocrit value decreased from 54% at diagnosis to 45% at 12 months, and adequate hematocrit control over time (< 45%) was observed in 36%, 44%, and 32% of the patients at 6, 12, and 24 months, respectively. More than 5 phlebotomies per year in the maintenance phase were required in 19% of patients. Worsening thrombocytosis, age > 60 years, and microvascular symptoms constituted the main indications for starting cytoreduction. Median duration without initiating cytoreduction was significantly longer in patients younger than 50 years (< 0.0001). The incidence rate of thrombosis under phlebotomies alone was 0.8% per year and the estimated probability of thrombosis at 10 years was 8.5%. The probability of arterial thrombosis was significantly higher in patients with arterial hypertension whereas there was a trend to higher risk of venous thrombosis in cases with high *JAK2V617F* allele burden. Rates of major bleeding and second primary neoplasm were low. With a median follow-up of 9 years, survival probability at 10 years was 97%, whereas the probability of myelofibrosis at 10 and 20 years was 7% and 20%, respectively. Progression to acute myeloid leukemia was documented in 3 cases (1%). Current management of low-risk PV patients is associated with low rate of thrombosis and long survival. New treatment strategies are needed for improving hematological control and, in the long term, reducing progression to myelofibrosis.

## Introduction

Polycythemia vera (PV) is a myeloproliferative disorder characterized by clonal erythrocytosis and increased risk of hemorrhagic and thrombotic events [1]. Risk-adapted therapy relies on the presence of two risk factors, with low risk defined as age < 60 years without thrombosis history and high risk by either the presence of age ≥ 60 years or thrombosis history [2]. Other factors such as leukocytosis or high *JAK2V617F* allele burden have been related with the risk of thrombosis and disease progression, but they are not included in risk stratification yet [3–6].

The main objective of therapy in low-risk PV is controlling symptoms and preventing thrombotic and hemorrhagic events. Standard first-line therapy for low-risk patients includes primary prophylaxis of thrombosis with low-dose aspirin and Hct control with phlebotomies. Although cytoreductive therapy might result in a higher reduction of thrombosis than phlebotomies alone, the use of hydroxyurea in low-risk PV patients is not recommended since the potential carcinogenic and teratogenic risk of this treatment could be greater than its benefit in reducing thrombosis [2, 7–12].

The recent approval of new agents, such as ropeginterferon and ruxolitinib, has changed the treatment scenario for PV [13–16]. Moreover, the potential disease-modifying effect of ropeginterferon has increased interest of this drug in low-risk patients, and new recommendations have recently been published to guide the indication for cytoreductive therapy [17]. However, the actual impact of phlebotomies in reducing thrombo-hemorrhagic complications in low-risk PV and their ability to achieve hematological and symptomatic control have not been extensively assessed.

The objective of this study was to evaluate the hematologic control, complications, and cytoreductive needs of a large series of 453 patients with low-risk PV from the Spanish Registry of PV. These new data will help to understand the real unmet needs of these patients and to position these new drugs in the therapeutic algorithm of the disease.

## Patients and methods

The Spanish Registry of Polycythemia Vera was initiated in July 2011 and is periodically updated. It is sponsored by GEMFIN (Grupo Español de Enfermedades Mieloproliferativas Filadelfia Negativas). By April 2021, a total of 2245 patients from 60 Spanish hospitals were included in the registry. Patients were eligible for inclusion in the present study if they had low-risk PV and were treated only with phlebotomies. Informed consent for the inclusion in this registry and the scientific use of these data was obtained in accordance with the requirements of the local ethics committees.

A total of 453 patients with low-risk PV diagnosed according to the World Health Organization (WHO) criteria were included. Low-risk PV was defined as age < 60 years old and no history of prior thrombosis. The phlebotomy indication was made by the treating hematologist with the objective to maintain the Hct below 45% according to ELN recommendations [2]. Primary prophylaxis of thrombosis with antiplatelet therapy, usually low-dose aspirin, was indicated in all patients unless contraindicated. All patients were treated in tertiary centers with weekly visits until Htc control and every 3–4 months during the second year and thereafter.

Disease-related symptoms including pruritus, microvascular disturbances and splenomegaly, hemoglobin (Hb), Hct, leukocyte count, and platelet count were assessed at PV diagnosis and at months 6, 12, 18, 24, 36 and 60 months while undergoing phlebotomy therapy alone. Date and reason for starting cytoreductive therapy; main disease complications including thrombosis, bleeding, disease progression, and second primary neoplasia; and status at last follow-up were recorded. Thrombosis was defined according to the International Classification of Diseases (ninth revision) including superficial thrombophlebitis. Severe hemorrhage was defined as a symptomatic bleeding in a critical organ, or an overt hemorrhage requiring transfusion or associated with an Hb decrease > 20 g/l without transfusion.

Survival, disease progression, thrombosis, hemorrhage, and second primary neoplasms during phlebotomy therapy were calculated. Time to first thrombotic event was calculated from diagnosis with patients being censored at the last visit or at the time of initiating cytoreduction. Survival and time to disease progression were calculated until last contact, including follow-up after cytoreduction initiation. Time to event curves were drawn by the method of Kaplan and Meier with the log-rank test for comparisons. Multivariate analysis was performed by the Cox regression. Statistical analyses were performed with SPSS, version 25. Relative survival was estimated by the cohort method as described by Dickman from life tables of all-cause mortality in the Spanish population, stratified by age, sex, and calendar year of diagnosis, that were obtained from the Human Mortality Database. Relative survival analysis was performed with Stata, v.14 (www.stata.com). The incidence rate of the main clinical outcomes was also calculated as the number of events/total follow-up in person-years.

## Results

### Main clinical and hematological values at diagnosis and during follow-up in low-risk PV patients managed with phlebotomies alone

The main clinical characteristics at diagnosis of PV are shown in Table 1. The median age was 49 years, and 256 patients (57%) were male. In addition to phlebotomies, concomitant antiplatelet therapy was used in 90% of patients. Microvascular symptoms were present in 172 patients (39%) at diagnosis, including erythromelalgia, headache, visual alterations, and paresthesia in 8%, 25%, 3%, and 8% of the patients, respectively.

**Table 1:** Main clinical characteristics at diagnosis in 453 low-risk patients from the Spanish Registry of Polycythemia Vera

Age, median (range)	49 (16–60)
< 50 years, *n* (%)	245 (54)
Male sex, *n* (%)	256 (57)
Cardiovascular risk factors, *n* (%)	
Smoking Diabetes mellitus Hypertension Hypercholesterolemia	133 (29) 26 (6) 115 (25) 68 (15)
Symptomatic burden, *n* (%)	
Minor hemorrhage Microvascular symptoms Pruritus	5 (1) 172 (39) 147 (33)
Hematological values, median (IQR)	
Hemoglobin, g/l Hematocrit, % Leukocyte count, × 10^9^/l Platelets, × 10^9^/l	176 (163–190) 54 (50–59) 9.4 (7.8–12) 478 (335–630)
Palpable spleen, *n* (%)	65 (15)
*JAK2* mutation, *n* (%) *JAK2*V617F, *n* (%) %VAF (median, extremes) Exon 12, *n* (%)	397* (96) 386 (97) 32 (16–75) 11 (3)

^*^Available in 414 of 453 patients. *IQR*, interquartile range; *VAF*, variant allele frequency

Persistence of microvascular symptoms under phlebotomy treatment was reported in 12%, 13%, 10%, 7%, and 13% of the patients at 12, 24, 36, 48, and 60 months of therapy, respectively. As for pruritus, it was present in 147 patients (33%) at diagnosis and persisted in 20%, 20%, 16%, 19%, and 25% of the patients at 12, 24, 36, 48, and 60 months of phlebotomy treatment, respectively. Spleen remained palpable in 2%, 5%, 8%, 7%, and 5% of the patients at months 12, 24, 36, 48, and 60, respectively, being symptomatic in half of them.

**Fig. 1:**
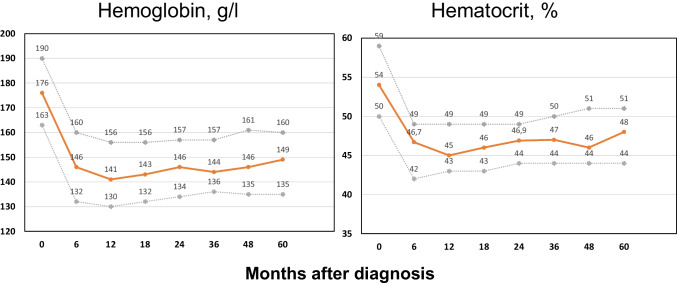
Hemoglobin level and hematocrit values over time in 453 low-risk patients managed with phlebotomies alone from the Spanish Registry of Polycythemia Vera. Medians (orange solid line) and interquartile ranges (gray dotted lines) at 6, 12, 18, 24, 36, 48, and 60 months of therapy are provided. Numbers of patients treated with phlebotomies alone were 369, 322, 284, 262, 220, 191, and 167, at months 6, 12, 18, 24, 36, 48, and 60, respectively. Hematological data were reported in 183, 148, 127, 124, 97, 87, and 83 patients at months 6, 12, 18, 24, 36, 48, and 60, respectively

Hemoglobin values and hematocrit levels over time are shown in Fig. 1. As can be seen, the median Hct value decreased from 54% at baseline to 46.7% and 45% in the assessments corresponding to 6 and 12 months, respectively. The percentage of patients with adequate hematocrit control over time (< 45%) was 36%, 44%, 36%, and 32% at 6, 12, 18, and 24 months after diagnosis, respectively. These figures decreased to 29%, 30%, and 30% at 3, 4, and 5 years, respectively.

Conventional phlebotomy was the most frequent procedure, while erythroapheresis was used in 8.5% of patients. Median whole blood volume extracted in each procedure was 450 ml for conventional phlebotomy and 440 ml of red blood cell concentrate for erythroapheresis. Complete data on the number of phlebotomy/erythroapheresis procedures during the entire observation period were available in 109 cases; the median number of phlebotomies was 6 (range: 2–26) in the first year of therapy and 3 phlebotomies per year (range: 1–11) during the second year and thereafter. More than 5 phlebotomies per year in the maintenance phase (after first year of therapy) were required in 19% of patients.

**Fig. 2:**
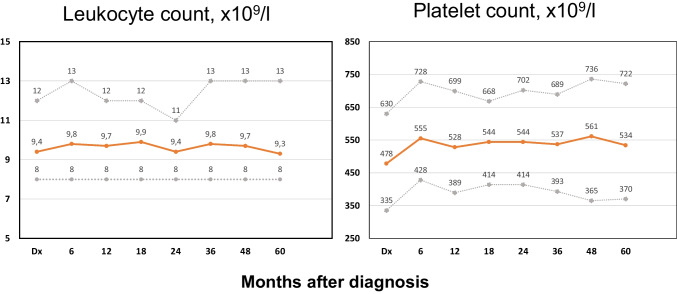
Leukocyte and platelet count over time in 453 low-risk patients managed with phlebotomies alone from the Spanish Registry of Polycythemia Vera. Medians (orange solid line) and interquartile ranges (gray dotted lines) at 6, 12, 18, 24, 36, 48, and 60 months of therapy are provided. Numbers of patients treated with phlebotomies alone were 369, 322, 284, 262, 220, 191, and 167 at months 6, 12, 18, 24, 36, 48, and 60, respectively. Hematological data were reported in 183, 148, 127, 124, 97, 87, and 83 patients at months 6, 12, 18, 24, 36, 48, and 60, respectively

Leukocytosis > 15 × 10^9^/l was present in 34 (7.5%) patients at diagnosis. Leukocyte counts over time are showed in Fig. 2. Median leukocyte counts remain stable under phlebotomies with leukocytosis > 15 × 10e9/l being reported in 11%, 11%, 15%, 13%, and 11% of the patients at months 12, 24, 36, 48, and 60, respectively. Platelet counts are shown in Fig. 2. As can be seen, median platelet count increased after starting phlebotomies with thrombocytosis > 1000 × 10^9^/l being reported 2.4% at diagnosis and in 4%, 2.5%, 4%, 6%, and 7% of the patients at months 12, 24, 36, 48, and 60, respectively. Extreme thrombocytosis (platelet count > 1500 × 10^9^/l) was not reported in any case at diagnosis or during follow-up.

### Duration of therapy with phlebotomies alone and reasons to start cytoreduction

**Fig. 3:**
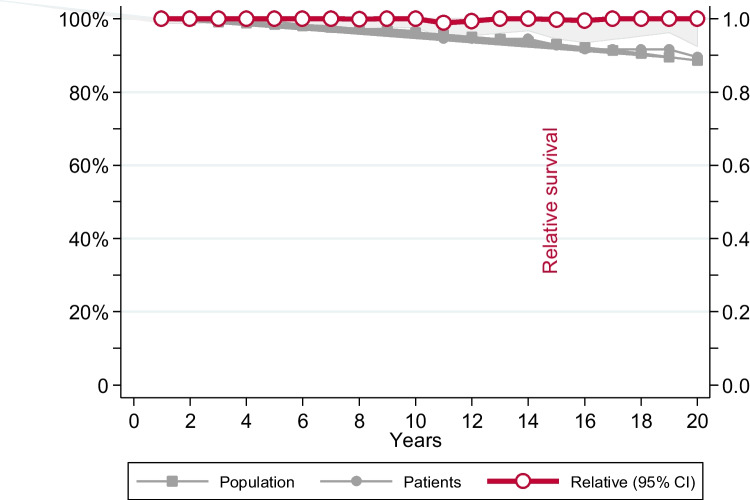
Actuarial survival estimated in 453 patients with low-risk polycythemia vera as compared with the expected survival in the matched general population, according to the period of diagnosis. Relative survival (red line) is displayed on the left *y*-axis

During follow-up, 272 out of 453 (60%) patients were initiated on cytoreductive treatment. Median time elapsed from diagnosis to cytoreduction start was 5.7 years (0.1–26). The main indications for starting cytoreduction are shown in Table 2. First-line cytoreductive treatments included hydroxyurea and interferon in 258 (57%) and 14 (3%) patients, respectively. The median duration of therapy with phlebotomy alone was significantly longer in patients younger than 50 years than in those older than 50 years (8.4 years versus 3 years, respectively, *p* < 0.0001) (Fig. 3).

**Table 2:** Main indications for starting cytoreductive therapy in 453 low-risk patients from the Spanish Registry of Polycythemia Vera

Thrombocytosis	72 (26.5)
Age > 60 years	54 (20)
Microvascular symptoms	50 (18)
Thrombosis	19 (7)
Inadequate hematocrit control with phlebotomies	20 (7)
Leukocytosis	9 (3)
Symptomatic splenomegaly	5 (2)
Pruritus	4 (1)
Bleeding	4 (1)
Iron deficiency	2 (0.4)
Other	18 (7)
Not reported	15 (5.5)
Total	272

Results are presented as number of patients (percentage)

### Survival and disease progression

**Fig. 4:**
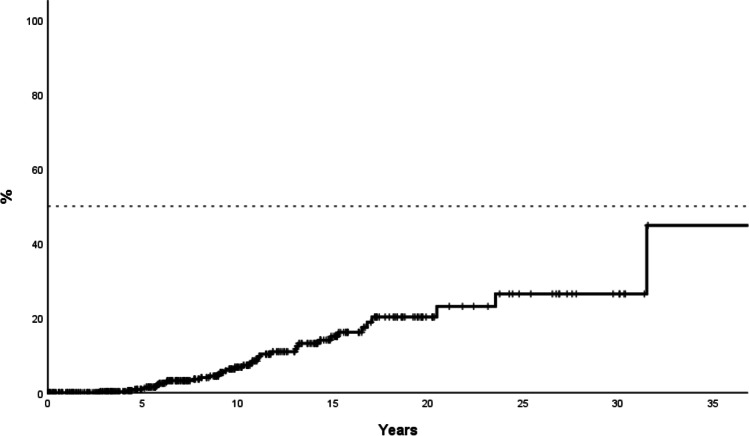
Probability of myelofibrosis in 453 low-risk patients initially managed with phlebotomies alone from the Spanish Registry of Polycythemia Vera. Progression to myelofibrosis at 10 and 20 years was 7% and 20% respectively. Patients were censored at last contact including follow-up after cytoreduction start

With a median follow-up of 9 years, 23 (5%) patients died resulting in an estimated mean survival of 33 years (95%CI: 30–36). The survival probability at 10 and 20 years was 97% and 92%, respectively. Relative survival (RS) with the matched general population of same age and sex is shown in Fig. 4. Patients died at the same rate as that of Spanish general population with the relative survival at 20 years being 1 (Fig. 3). Causes of death included the following: disease progression *n* = 4, thrombosis *n* = 3, major bleeding *n* = 1, infection *n* = 4, second primary neoplasia *n* = 3, heart failure *n* = 2, other *n* = 2, not reported *n* = 2. Median age at time of death was 64 years (range: 25–86), with 8 patients being younger than 60 years. Only three patients were receiving phlebotomies alone (in addition to the standard antithrombotic prophylaxis) at time of death. In these cases, the causes of death were massive pulmonary embolism, myocardial infarction, and not reported.

Progression to MF occurred in 37 (8%) patients, 8 of them under therapy with phlebotomies alone and the remaining after cytoreduction initiation. The probability of myelofibrosis at 10 and 20 years was 7% and 20%, respectively (Fig. 4). The probability of myelofibrotic transformation at 20 years was 14% and 30% in patients younger and older than 50 years, respectively (*p* = 0.004). The *JAK2*V617F allele burden was available in 237 patients, and it was associated with a trend towards higher probability of myelofibrotic transformation (20-year probability: 4.7% and 33% in patients with low (< 50% VAF) and high (≥ 50% VAF) *JAK2V617F* allele burden, respectively, *p* = 0.08). On multivariate analysis, age > 50 years (HR: 2.5, 95%CI: 0.97–6.6, *p* = 0.06) and high *JAK2V617F* allele burden (HR: 2.06, 95%CI: 0.8–5.08, *p* = 0.12) at diagnosis were associated with a non-significant trend to higher risk of myelofibrosis.

Progression to AML was documented in 3 cases (1%). Age at time of acute transformation was 57, 64, and 65 years. Elapsed time from diagnosis to acute leukemia was 6 years in two cases and 9 years in one case. One of the patients had been treated with hydroxyurea for 3 years due to progressive leukocytosis and thrombocytosis under phlebotomies whereas the remaining two had not been exposed to cytoreductive therapy before AML.

### Thrombosis, bleeding, and second primary neoplasm under phlebotomies

**Fig. 5:**
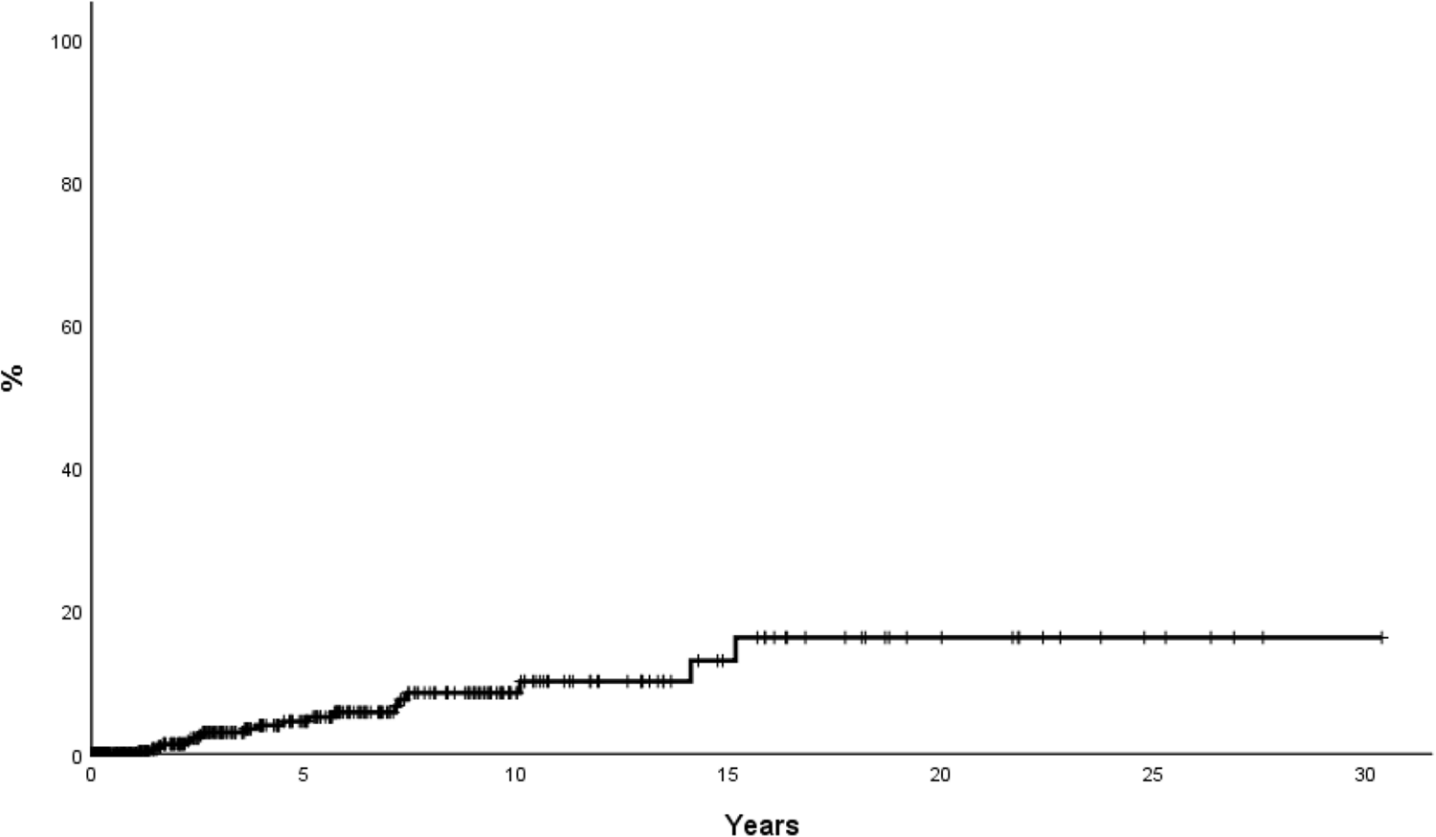
Time to first thrombotic event thrombosis under therapy with phlebotomies alone in 453 low-risk patients initially managed with phlebotomies alone from the Spanish Registry of Polycythemia Vera. The estimated probability of thrombosis at 10 years was 8.5%. Patients without thrombosis were censored at time of cytoreduction start or last contact

A total of 19 patients experienced a thrombotic event while on therapy with phlebotomies alone (12 arterial and 7 venous). With a 2239 person-years follow-up, the incidence rate of thrombosis was 0.8% per year and the estimated probability of thrombosis at 10 years was 8.5% (Fig. 5). The probability of arterial thrombosis at 10 years was 5.7%. Arterial hypertension was associated with a higher probability of arterial thrombosis in univariate analysis (11.7% versus 3.9% in patients with and without arterial hypertension, respectively, *p* = 0.02). On multivariate analysis, arterial hypertension showed a tendency towards a higher risk of arterial thrombosis (HR: 2.98, 95%CI: 0.90–9.8, *p* = 0.07) after adjustment for age and sex. The probability of venous thrombosis at 10 years was 3%, being higher in patients with high *JAK2V617F* allele burden (12% and 2.5% for patients with high and low allele burden, respectively, *p* = 0.058). On multivariate analysis, *JAK2* allele burden showed a non-significant trend to higher risk of venous thrombosis (HR 4.86, 95%CI: 0.9–26.3, *p* = 0.067). Age at diagnosis, sex, smoking, diabetes, hypercholesterolemia, and hematological values at diagnosis, including leukocyte count, platelet count, and hemoglobin level, were not associated with the probability of total thrombosis, arterial thrombosis, or venous thrombosis during phlebotomy treatment.

Bleeding was reported in 12 (2.6%) patients, corresponding to major and minor bleeding in 4 and 8 patients, respectively. The rate of major bleeding was 0.18% per year. Second primary neoplasia was reported in 7 patients (0.3% per year) including 2 cases of colon carcinoma and one case each of renal carcinoma, bladder carcinoma, liposarcoma, melanoma, and angioma.

## Discussion

There are few studies focusing on hematological and symptomatic control, incidence of complications, and need for cytoreduction in low-risk PV patients treated with phlebotomies. The present study included 453 low-risk patients from the Spanish Registry of PV who were initially treated only with phlebotomies. Notably, the median Hct value decreased from 54% at baseline to 45% at 12 months, but a sustained hematocrit < 45% was achieved only in third of patients. In addition, a significant proportion of patients persisted with PV-related symptoms, splenomegaly, leukocytosis, and/or thrombocytosis under phlebotomies, and in some of them, this led to indication for starting cytoreduction. Therefore, our data demonstrate that symptomatic and hematological control is not fully achieved in a proportion of patients treated with phlebotomies alone, providing the basis for initiating cytoreduction in low-risk PV patients. According to recent ELN recommendations (17), cytoreductive drugs should be considered in patients with persistent leukocytosis (> 15 × 10^9^/l), extreme thrombocytosis (> 1500 × 10^9^ platelets per l), or inadequate hematocrit control with phlebotomies (a need for at least six phlebotomies per year for at least 2 years in the maintenance phase). If we apply these criteria to our series, the proportion of low-risk patients treated with phlebotomies who might be candidates for cytoreduction due to persistent leukocytosis, extreme thrombocytosis, and inadequate Hct control would be 11%, 0%, and 19%, respectively.

Scherber et al. reported higher symptomatic burden in patients treated with phlebotomy alone than in those who received cytoreduction, highlighting the need for a correct symptom assessment in patients treated with phlebotomies [18]. In this regard, microvascular symptoms and pruritus were frequently reported in our series, but due to the retrospective study design, the severity of these symptoms was not assessed using the MPN-SAF or other standardized symptom scores, which prevents us from estimating what proportion of these patients might benefit from a trial of cytoreductive therapy.

The incidence of thrombotic events in our cohort of low-risk PV patients while receiving treatment with phlebotomies alone was 0.8% per year. The IWG-MRT [3], ECLAP [10], and CytoPV [11] studies reported rates of 2.62%, 4.4%, and 2.7% thrombotic events per year, respectively, but these studies included low- and high-risk PV patients. In fact, most of the events reported in patients treated with phlebotomies alone in the ECLAP study occurred in high-risk patients, with few thrombotic events reported in low-risk patients [10]. Overall, this low incidence of thrombosis is remarkable, considering that only 30–40% of patients in the ECLAP study and in our series had adequate hematocrit control over time [10]. These figures are in line with the Low-PV study in which the superiority of ropeginterferon alfa-2b over phlebotomies alone has been demonstrated, with hematocrit control in 66% of patients treated with phlebotomies under strict monthly hematological assessment [19]. Whether therapeutical intervention with cytoreduction could further reduce the incidence of thrombotic events in this low-risk population is unknown. Of note, a recent meta-analysis has reported a frequency of 0.5% thrombotic events per year in PV patients treated with interferon, a figure similar to that reported in the general population [20]. However, the low frequency of thrombotic events in this patient population makes difficult to show a benefit in primary prevention of thrombosis with conventional clinical trials.

In our series, the estimated median survival was longer than 30 years with more than 90% of patients being alive at 10 years from diagnosis. Only three deaths were reported under phlebotomies alone, two of them due to thrombotic complications reinforcing the need of adequate thrombotic prevention in this subset of patients. Relative survival analysis showed no reduction in expected survival when compared with the Spanish general population of same age and sex. This result should be interpreted with caution since the progression of the disease, especially to MF, could produce a reduction of survival in long-term survival not detected in our study. This is especially relevant for young patients and highlights the need of new agents with a disease-modifying effect for low-risk PV.

Although the present study constitutes a good representation of real practice in patients with low-risk PV managed with phlebotomies, several limitations should be considered when interpreting the results. The multicentric nature of the study is inevitably associated with differences in phlebotomy procedures, including the technique employed, the frequency of hematologic controls, or the total volume removed. It should be noted that no Hct evaluations were collected before month 6 precluding us to estimate the median elapsed time from diagnosis to Hct control under phlebotomies. In addition, hematological values during follow-up were not available in a significant proportion of patients, which precluded analysis of the association between hematocrit control and risk of thrombosis. Nevertheless, and despite its retrospective design, the present study constitutes a good representation of real-life clinical practice in a large series of low-risk PV patients with long follow-up.

We concluded that in low-risk PV patients managed with phlebotomies alone, the incidence of thrombosis and hemorrhage is low despite suboptimal hematological and symptomatic control. Progression to myelofibrosis constitutes an unmet need deserving the development of new treatment strategies.
